# Real-time processing of misinformation and its correction: Insights from eye movements during reading

**DOI:** 10.1186/s41235-026-00723-z

**Published:** 2026-03-22

**Authors:** Roslyn Wong, Lili Yu, Aaron Veldre, Erik D. Reichle

**Affiliations:** 1 School of Psychological Sciences, Macquarie University, Sydney, NSW 2109 Australia; 2 Graduate School of Health, University of Technology Sydney, Sydney, Australia

**Keywords:** Continued-influence effect, Eye movements, Individual differences, Misinformation, Reading comprehension

## Abstract

People often continue to rely on information even after it has been retracted–a phenomenon known as the *continued-influence effect* (*CIE*) of misinformation. This study investigated real-time indicators of misinformation susceptibility by recording the eye movements of 74 participants as they read pairs of newspaper-style articles containing critical information about the cause of an event that was either retracted or not. A post-reading questionnaire assessed memory for the passages and inferential judgements related to the retracted information. The roles of individual differences in language proficiency and working memory on the CIE were also tested. Questionnaire data replicated prior findings that repetition of the original information improved recall memory for the event. Eye-tracking data revealed that retractions were associated with increased processing effort during encoding of corrective information and reduced re-reading of non-causal details. Reminders of the original misinformation were linked to faster overall reading speeds. Higher reading proficiency predicted greater reductions in misinformation susceptibility, and both reading proficiency and verbal working memory capacity facilitated real-time processing of causal information. Finally, longer reading times and slower reading speeds were tentatively associated with reduced misinformation susceptibility but only when retractions were presented without explicit reminders. Together, these findings suggest that misinformation susceptibility reflects both individual differences in cognitive abilities and the effectiveness of reminder-based corrections.

## Significance Statement

Misinformation is an ongoing challenge to informed decision-making around the world, particularly in critical domains such as public health, politics, and climate change mitigation. One compelling example of the power of misinformation is the *continued-influence effect* (CIE), whereby individuals often continue to rely on information even after it has been explicitly retracted and identified as false. Despite the importance of understanding and combatting the impact of misinformation, most CIE research has focused primarily on post-exposure comprehension and has largely overlooked the real-time cognitive processes that unfold as individuals initially encounter false information. The present research addressed this gap by recording eye movements as participants read and processed written accounts of an event containing causal information that was subsequently retracted. The results showed that readers were sensitive to corrections of causal information, which were associated with increased processing effort. Moreover, repeating the original misinformation alongside its correction led to faster reading speeds, suggesting that explicit reminders facilitated the integration of updated information. Higher reading proficiency and verbal working memory capacity also predicted more effective integration of corrective information. These findings deepen our theoretical understanding of the CIE and provide evidence for the efficacy of reminder-based correction strategies in reducing misinformation susceptibility.

## Introduction

*Misinformation* refers to any type of information that is false or inaccurate, regardless of its source or intent (Wu et al., 2019). While some misinformation is relatively benign, such as the myth that *lightning never strikes the same place twice*, other types of misinformation have the potential to cause more widespread damage by increasing misperceptions and resistance to knowledge, such as the claim that *the MMR vaccine causes autism*. Advances in digital technology have dramatically increased the creation and dissemination of fake and misleading information, and it is widely acknowledged that misinformation does and will continue to pose an ongoing challenge for individuals and societies (O’Connor & Weatherall, 2019). Indeed, over the past decade alone, the spread of misinformation has had negative consequences for public health, the implementation of positive environmental behaviors, and maintaining stable democracies (Lewandowsky et al., 2017; Loomba et al., 2021). It is therefore increasingly important to understand the causes and consequences of individuals’ beliefs in misinformation so that effective interventions can be implemented.

An example of the power of misinformation is a phenomenon known as the *continued-influence effect* (*CIE*), where information that is initially presented as true but later explicitly retracted and identified as false continues to influence individuals’ memory, inferential reasoning, and decision making (Johnson & Seifert, 1994; Wilkes & Leatherbarrow, 1988). In the laboratory, the CIE is typically demonstrated via a text comprehension task in which participants are presented with details of an event (e.g., a fire) that includes a critical piece of information (e.g., “the fire was deliberately lit”) which is either retracted or not. Understanding of the event is probed via a series of post-reading questions (e.g., “Would it be appropriate to punish someone for the fire?”). The typical finding is that individuals continue to report and rely on the original information even when they acknowledge and remember the retraction (Ecker et al., 2011a; Ecker et al., 2011b; see Lewandowsky et al., 2012; Walter & Tukachinsky, 2020 for reviews). This effect has been reliably induced and has proven difficult to eliminate across a variety of contexts, including real-world news (Lewandowsky et al., 2005), common myths (Swire et al., 2017), and even when using subtle or implied information (Ecker et al., 2014).

Because of the real-world implications of the CIE, a substantial body of research has focused on designing effective correction strategies to reduce misinformation susceptibility (see Prike & Ecker, 2023 for a review). One strategy that has been found to be effective is the repetition of the original information alongside its retraction (see Kemp et al., 2024a for a review)—for example, in the case of the hypothetical fire, a reminder of the cause that was originally reported (i.e., arson) when later correcting the cause of the fire. This *reminder strategy* is hypothesized to reduce misinformation reliance because it highlights the discrepancy between the two causal explanations provided by reinstating the original information alongside its correction (Ecker et al., 2017; Kendeou et al., 2014; Putnam et al., 2014). This makes it more likely that the misinformation will be subsequently updated with the correct information. Consistent with this account, Ecker et al. (2017) demonstrated that retractions accompanied by an explicit reminder of the original critical information led to decreases in the CIE relative to retractions without a reminder (see also Kemp et al., 2022, 2024b; Wahlheim et al., 2020, 2025). A closely related study by Xu et al. (2020) found that this reminder effect generalized across print and mobile reading formats, and across English- and Chinese-speaking participants. Although this reminder-based correction strategy might be expected to inadvertently increase the perceived truth of misinformation via a *familiarity backfire effect* (Lewandowsky et al., 2012; Pluviano et al., 2017; Swire et al., 2017; see also Begg et al., 1992; Dechêne et al., 2010; Schwarz et al., 2007), the available evidence instead suggests that reminders are associated with *reductions* in the CIE, providing little support for this alternative account (see Swire-Thompson et al., 2020, 2022 for reviews).

### Using eye-tracking to investigate the CIE

Despite growing research on the CIE over the past decade, most studies have relied on self-report questionnaires comprising open- and close-ended questions. Although questionnaires are effective for measuring the consequences of misinformation, they capture only the offline product of comprehension *after* exposure to false information. In other words, the measures provide limited insight into *how* misinformation is processed *at the moment* it is encountered. A complementary online method is therefore needed to assess the real-time processing of misinformation and to evaluate the effectiveness of correction strategies, such as eye-tracking.

During reading, individuals move their eyes from one word to the next through an alternating pattern of *fixations*, or intervals when the eyes remain stationary on a word, and *saccades*, or eye movements. This behavior reflects the overt allocation of attention and is widely assumed to index the underlying cognitive processes supporting text comprehension (Hyönä et al., 2003), under the assumption that the eyes remain fixated on a word for the duration of its cognitive processing (Rayner, 1998). Thus, increasing processing demands by manipulating features of a text (e.g., word frequency or contextual constraint) reliably modulates eye-movement measures, such as fixation durations and the number of *regressions,* or backward saccades in the text (Rayner et al., 2006). Individual differences among readers are also reflected in eye-movement behavior, including differences in reading ability (Ashby et al., 2005; Perfetti, 2007), prior knowledge (McNamara & Magliano, 2009), and memory capacity (Daneman & Carpenter, 1980). Eye-tracking thus captures the cognitive and linguistic processes that support text comprehension (Rayner, 1998, 2009). Given that many instances of misinformation are encountered in written form, eye-tracking during reading is one non-invasive method for gaining real-time insight into how text-based misinformation is processed and how effectively it can be corrected.

To date, only a handful of studies have used eye-tracking to investigate misinformation effects during reading. Some have compared eye-movement behavior when processing information (e.g., news) that is either real or fake, typically finding that fake information yields longer total viewing times and more regressive saccades than real information (Bozkir et al., 2022; Sümer et al., 2021). More directly relevant to the present research are studies that have examined eye-movement behavior when misinformation is subsequently corrected. For example, Kim et al. (2021) presented misinformation about the HPV vaccine in the form of a tweet that was subsequently corrected with additional text, either accompanied by a humorous or non-humorous image. While they observed no differences in eye movements to the correction text, humorous images yielded longer total viewing times compared to non-humorous images. Similarly, Lee et al. (2024) used a Facebook newsfeed paradigm and reported longer total viewing times for regions containing factual elaborations that corrected food-crisis-related misinformation compared to simple rebuttals. Finally, Wellons and Wahlheim (2025) presented false headlines that were corrected with or without a misinformation reminder, or not corrected at all, and found that key details that determined the veracity of the headline were fixated more quickly when a reminder-based correction was present. Taken together, these studies converge in showing that readers are sensitive to the presence of misinformation which alters their eye-movement behavior in response to corrective information. Further, regardless of the modality used to correct misinformation, individuals tend to direct their attention towards and increase their encoding of this information. However, although these eye-movement studies are important contributions, the findings should be interpreted with caution for several reasons.

First, these studies generally relied on a restricted set of global eye-movement measures, potentially limiting insight into the cognitive processes underlying misinformation effects. Local eye-movement measures reliably map onto different stages of processing—early measures (e.g., first-fixation durations) reflect lexical processing while late measures (e.g., regressions) are typically linked to the effort required to integrate a word into its sentence representation (Clifton et al., 2007; Vasishth et al., 2013). However, most of the studies reviewed above reported only total fixation counts or total viewing times, which are global summary measures that provide a coarse index of the processing difficulty associated with an entire text and do not distinguish between early and late stages of processing (but see Wellons & Wahlheim, 2025, for the use of time bins). Furthermore, these measures were often extracted from imprecisely defined regions of interest (e.g., a rectangular display; Kim et al., 2024). These limitations make it difficult to localize observed effects to specific manipulations or to rule out the contribution of processes unrelated to reading the misinformation or its correction. To the extent that prior studies failed to examine local eye-movement measures on specific regions of interest, their conclusions may have oversimplified the cognitive mechanisms underlying misinformation effects.

Second, the eye-movement studies reviewed above examined misinformation effects using multimodal materials comprising both text and images. Although visual information can influence how text is understood, it remains unclear whether images, on average, facilitate or hinder comprehension. Images are often intended to support understanding (Messaris & Abraham, 2001), and have been argued to enhance text credibility and persuasiveness (Hameleers et al., 2020; Powell et al., 2015). However, this may come at a cost because the attention required to encode and process multimedia text must be divided across multiple sources (Liao et al., 2024). Any benefits of multimodal information may be offset by reduced attention to the text. Given these complexities, it is important to investigate misinformation effects and the effectiveness of correction strategies using textual materials without competing visual information.

Finally, the relationship between individuals’ eye-movement behavior and their susceptibility to misinformation has not been systematically examined in prior research. Existing studies have either failed to examine this relationship (e.g., Lee et al., 2024) or have done so only indirectly (e.g., Kim et al., 2021; Wellons & Wahlheim, 2025). For example, Kim et al. (2024) found that increased viewing time of corrective information indirectly reduced subsequent misperceptions about the HPV vaccine by reducing the perceived credibility of the original incorrect information. Similarly, Wellons and Wahlheim (2025) found that earlier and more frequent fixations on corrective details predicted participants’ confidence in whether headlines had been corrected, but they did not assess whether there were any ongoing effects of the original misinformation. Thus, it remains unclear whether eye-movement measures during the processing of misinformation can directly predict later susceptibility to the continued influence of retracted information. Demonstrating such a link would indicate that the allocation of attention during misinformation processing has downstream consequences for the persistence of false or inaccurate beliefs.

### Individual differences in the CIE

Although correction strategies have been designed to combat misinformation susceptibility, these strategies often attribute the CIE to the inherent misunderstanding of presented facts, and do not consider the underlying factors that may render misinformation resistant to correction. These factors include the potential role of individual differences in cognitive abilities.

A potentially important factor is individual differences in reading proficiency. Skilled readers—individuals who can efficiently coordinate the simultaneous demands of word identification and text comprehension—are better able to construct veridical and coherent mental representations or *situation models* of what is being described in text (Kintsch, 1998; Zwaan & Brown, 1996; Zwaan & Radvansky, 1998). Because the CIE paradigm typically involves written materials, a reader’s ability to revise a fact or scenario following a retraction may depend on their general decoding and comprehension skills. Individuals with higher reading proficiency should therefore be more skilled at comprehending complex scenarios compared to individuals with poorer reading proficiency. Consistent with this view, the only two studies to examine this relationship found that higher reading proficiency was associated with reduced susceptibility to the continued influence of retracted information (De Keersmaecker & Roets, 2017; Xu et al., 2020). However, these studies relied on relatively simple measures of reading proficiency: De keersmaecker and Roets used a short 10-item vocabulary subtest from the Wechsler Adult Intelligence Scale, while Xu et al. combined the 40-item Shipley Vocabulary Scale (Shipley, 1940) and Author Recognition Test (Stanovich & West, 1989), which indexes reading experience. Further research is therefore needed to examine the relationship between misinformation susceptibility and reading proficiency using measures that capture a wider range of reading skills.

Another factor that may influence the CIE is individual differences in *working memory* (*WM*)—the limited-capacity system responsible for storing and manipulating information (Baddeley, 1986, 2000; Oberauer, 2009). One possibility is that the CIE reflects difficulty integrating a correction with the original information in WM. As such, limited WM capacity may result in retracted information being poorly integrated and memory for an event not being accurately updated, allowing the misinformation to persist and thereby influence cognition (Johnson & Seifert, 1994; Wilkes & Leatherbarrow, 1988). Under this *integration* account, WM capacity delimits how much task-relevant information can be actively maintained and processed, which is then predictive of misinformation susceptibility. In the context of the CIE, however, empirical support for this relationship has been mixed. Some studies report that higher WM capacity is associated with reduced CIE susceptibility (e.g., Brydges et al., 2018), while others find no evidence of an association (e.g., Sanderson et al., 2021). These inconsistencies could be taken to support an alternative *retrieval* account of the CIE, which posits that, even when a retraction is present in memory, the original misinformation is selectively retrieved due to automatic familiarity processes (Ecker et al., 2010b; Lewandowsky et al., 2012; Swire et al., 2017). Further research is therefore needed to clarify the relationship between misinformation susceptibility and WM capacity, given its implications for theoretical accounts of the CIE.

### The present study

The present research had two primary aims. First, this study used eye-tracking to examine how misinformation is processed during reading and to assess the effectiveness of correction strategies. In addition to global reading measures that index overall processing difficulty across an entire text, local early and late reading measures were examined to track the time course of misinformation processing on specific regions of interest. Second, this study examined whether reading proficiency and WM capacity modulate sensitivity and susceptibility to misinformation.

To address these aims, the design of Ecker et al. (2017) was adapted by presenting participants with a series of passages containing information initially stated as true but subsequently retracted and identified as false while eye movements were recorded. To assess reductions in the CIE, the original misinformation was either repeated alongside the retraction or omitted. Participants’ comprehension was subsequently assessed using an offline questionnaire. Consistent with previous findings, it was expected that retractions, particularly those accompanied by a reminder, would reduce the CIE, as indexed by questionnaire responses. On eye-movement measures of reading, retractions were expected to elicit increased processing effort, reflected in longer and more frequent fixations at both the passage level and within regions associated with encoding corrective information. Individual differences were also expected to moderate misinformation effects, with lower CIE susceptibility emerging in individuals with higher reading proficiency and higher WM capacity. Finally, if online processing is associated with subsequent misinformation susceptibility, eye-movement measures were expected to predict the CIE.

## Method

### Participants

Eighty-eight undergraduate students from Macquarie University participated in the study in exchange for course credit. Data from three participants were excluded due to eye-tracker calibration difficulty. An additional 11 participants were excluded because data from one or both individual differences tasks were missing or indicated subpar performance, as detailed below. The final sample therefore comprised 74 participants (56 females, *M*_age_ = 20.51 years, range = 17–52 years).

Power analyses conducted using 100 Monte Carlo simulations with the *simR* package (Green & MacLeod, 2016) in *R* (Version 4.4.0; R Core Team, 2019) based on effect sizes reported by Ecker et al. (2017) indicated that a minimum of 19 participants was sufficient to detect the retraction effect (0.25 points) and reminder effect (0.12 points) with > .80 power. Additionally, following prior eye-movement studies of misinformation (e.g., Wellons & Wahlheim, 2025), a minimum sample size of 54 participants was targeted. All participants were native English speakers with normal or corrected-to-normal vision and provided written informed consent prior to participation. The study was approved by the Macquarie University Human Research Ethics Committee.

### Materials and design

#### Scenarios

Participants read 12 scenarios, half of which were taken directly from Ecker et al. (2017). Each scenario consisted of two short newspaper-style passages (49–155 words) describing an unfolding news event (e.g., a fire). The first passage was identical across conditions and contained a critical piece of causal information (e.g., that the fire was deliberately lit), which served as the potential target of a retraction. The second passage provided additional information about the event that varied across three conditions. In the *no-retraction* (*NR*) control condition, the critical information from the first passage was neither retracted nor repeated. In the *retraction-with-no-reminder* (*RNR*) condition, the critical information from the first passage was contradicted by alternative information that did not explicitly refer back to the original claim (e.g., “After a full investigation and review of witness reports, authorities have concluded that the fire was set off by lightning strikes.”). In the *retraction-with-explicit-reminder* (*RER*) condition, the same alternative information was presented alongside an explicit reminder of the original critical information (e.g., “It was originally reported that the fire had been deliberately lit, but authorities have now ruled out this possibility. After a full investigation and review of witness reports, it has been concluded that the fire was set off by lightning strikes.”).

The 12 scenarios were presented across three blocks of four scenarios each: two NR scenarios and one scenario in each retraction condition. Following previous studies (Ecker et al., 2017; Xu et al., 2020), scenarios within each block were presented in a fixed order to minimize participants’ awareness of retracted information during the reading task. The first item was always an NR scenario, and the two retraction conditions were separated by a second NR scenario. That is, the presentation order was always either: (i) NR, RNR, NR, RER or (ii) NR, RER, NR, RNR. Scenarios were counterbalanced across four lists such that, across participants, each scenario appeared approximately equally often in each condition and in each presentation order.

#### Questionnaire

Participants’ memory and comprehension of the scenarios were assessed using a questionnaire adapted from Xu et al. (2020), which was a shortened version of the original questionnaire developed by Ecker et al. (2017). For each scenario, two open-ended free-recall questions assessed participants’ memory (“Briefly summarise the “bushfire” scenario.”, “What was the cause of the bushfire?”), as well as three four-alternative multiple-choice questions (e.g., “Where did the bushfire occur?”). Three rating-scale questions assessed inferential judgments pertaining to the critical information (e.g., “Would it be lawful for someone to be punished as a result of the bushfire?”).

#### Individual differences tasks

All participants completed a battery of individual differences measures. Reading proficiency was assessed using the vocabulary and passage comprehension subtests of the Nelson Denny (*ND*) Reading Test (Brown et al., 1993) administered under half-time constraints to yield more normally distributed scores in samples of skilled readers (Andrews et al., 2020). The vocabulary subtest comprised 80 items (*α* = .91) and the passage comprehension subtest comprised 38 items (*α* = .87). Mean performance was 65% (*SD* = 13%) on the vocabulary subtest and 43% (*SD* = 17%) on the passage comprehension subtest. Because scores on the two subtests were highly correlated (*r* = 0.61, *p* < .001), standardized scores were averaged to create a composite reading proficiency index.

WM capacity was assessed using two adaptive complex span tasks: reading span (*RS*) and symmetry span (*SS*), which index verbal and nonverbal WM capacity, respectively (Gonthier et al., 2018). In the RS task, participants were visually presented with sequences of 2–8 letters, with each letter preceded by a sentence (e.g., “Birds are plants.”) requiring a TRUE/FALSE evaluation. At the end of each trial, participants recalled the letters in serial order, and RS scores reflected the total number of correctly recalled letters. In the SS task, participants viewed sequences of 2–8 squares presented in a 4 × 4 matrix, with each square preceded by an abstract black and white image that required a judgment of vertical symmetry. Participants then recalled the locations of the squares in serial order, and SS scores reflected the total number of correctly recalled squares. In both tasks, an adaptive procedure dynamically adjusted trial difficulty throughout the task based on performance.

Partial-credit load scoring was used for both WM tasks, in line with recommendations by Conway et al. (2005) and following prior studies (e.g., Brydges et al., 2018; Sanderson et al., 2021). Data from four participants were excluded because accuracy on the sentence evaluation component of the RS task or the image judgment component of the SS task was below 70%, indicating they were focusing only on the recall component in each task. Mean RS performance was 83% (*SD* = 8%) and mean SS performance was 79% (*SD* = 10%). Spearman-Brown split-half reliability estimates across sets were lower than typically observed for both RS (.31) and SS (.35), likely reflecting the fact that different set sizes were used dynamically across the tasks. Scores on the RS and SS tasks were moderately correlated (*r* = 0.28, *p* = .02).

### Apparatus

Participants’ eye movements were recorded using an SR Research EyeLink 1000 Plus eye-tracker as they read the scenarios on an HP X27q QHD monitor (1,920 × 1,080 resolution; 165 Hz refresh rate). Scenarios were presented across multiple lines in 18-pt black Consolas font on a grey background. Participants were seated 110 cm from the monitor with a chin and forehead rest used to minimize head movements. At this viewing distance, one degree of visual angle corresponded to approximately 4.32 letter spaces. Viewing was binocular, and eye movements were recorded from the right eye.

### Procedure

The experiment began with a practice trial to familiarize participants with the format of the scenario presentation and comprehension requirements. Participants were then randomly assigned to one of four counterbalanced lists, each comprising three blocks of four scenario. Each scenario consisted of two passages, which were always presented on separate display screens.^1^ Following Ecker et al. (2017), each passage was presented for a fixed maximum duration of 0.35 s per word to control overall reading time. This duration afforded a comfortable but not excessive amount of time to read each passage. Participants could progress to the next passage before the allotted time elapsed if they chose.

Each block began with a nine-point eye-tracker calibration procedure. If the maximum calibration error exceeded 0.5° of visual angle, calibration was repeated. Before each trial, a fixation point appeared at the location of the first letter of the passage. A stable fixation on this point was required before the passage was displayed. This initial fixation was not included in any analyses.

Following each block, a set of questionnaires related to the scenarios from that block was presented in the order in which the scenarios had appeared. Following Ecker et al. (2017), participants were given up to 5 min to complete the questionnaire for each scenario. Finally, all participants completed the battery of individual differences tasks.

The materials, data, and analysis code for this experiment are publicly available on the Open Science Framework website: https://osf.io/2qxbw/.

## Results

### Questionnaire scoring

Questionnaire responses were coded following the procedures used by Xu et al. (2020). Five dependent variables (described below) were coded independently by two scorers using the same criteria, with ambiguous cases resolved through discussion (see Table 1 for summary data).

**Table 1:** Mean (and standard deviation) scores on the questionnaire measures by condition

	NR	RNR	RER
General memory	0.56 (0.07)	0.54 (0.09)	0.55 (0.09)
Critical cause	0.73 (0.25)	0.53 (0.27)	0.65 (0.27)
Alternative cause	-	0.65 (0.24)	0.77 (0.24)
Retraction	-	0.32 (0.22)	0.53 (0.27)
Inference	0.70 (0.13)	0.55 (0.13)	0.49 (0.12)

A *general memory* score was computed for each scenario based on two components: (i) the number of correctly recalled idea units in participants’ responses to the open-ended question requiring them to summarize details of the scenario (maximum of four), and (ii) the number of correctly answered multiple-choice questions (maximum of three). These components were summed and averaged to form a score ranging from 0 to 1, where 1 indicated perfect recall of the scenario details. The coded idea units never referred to the original critical information or the alternative causal information.

*Memory for causal information* was assessed using three recall scores based on participants’ responses to the open-ended question requiring them to explicitly recall the cause of the event. Separate scores (0 or 1) were assigned for mentions of: (i) the original *critical cause* presented in the first passage (e.g., the fire was deliberately lit); (ii) the *alternative cause* presented in the second passage in the RNR and RER conditions (e.g., the fire was caused by lightning strikes); and (iii) the *retraction* or change in causal information (e.g., it was initially thought that the fire was deliberately lit, but it turns out it was caused by lightning strikes).

An *inference score* was calculated for each scenario based on participants’ responses to the three rating-scale questions assessing judgments pertaining to the critical information. These ratings were averaged and converted to a score ranging from 0 to 1, where higher scores indicated greater susceptibility to misinformation, i.e., the original critical information (e.g., stronger endorsement for “The government should spend more resources to prevent arson.”).

### Questionnaire analyses

Questionnaire data were analyzed using linear or logit mixed-effect models (LMMs/GLMMs) fit with the *lme4* package (Version 1.1–35.5; Bates et al., 2015) in *R*. Models tested the fixed effect of condition. For all dependent variables except the alternative cause and retraction scores, condition was coded using two orthogonal contrasts: (i) the *retraction effect*—the difference between the NR control condition and the average of the two retraction (RNR and RER) conditions, and (ii) the *reminder effect*—the difference between the RNR and RER conditions. For the alternative cause and retraction scores, which could not be computed for the NR condition, condition was coded as the difference between the RNR and RER conditions (i.e., the reminder effect).

To examine the role of individual differences, reading proficiency and verbal and nonverbal WM capacity were included as continuous, mean-centered predictors and allowed to interact with the condition contrasts. Maximal random-effects structures (i.e., subject and item random intercepts and slopes for the condition effect) either failed to converge or showed singular fits. Random-effects structure were therefore simplified by sequentially removing random slopes accounting for the least variance until model convergence without singular fit. Estimates yielding *t*/*z* values greater than |1.96| were interpreted as significant at the .05 *α* level. Full model outputs are provided in Table 2.

**Table 2:** Results of the (G)LMMs for the questionnaire measures

	Fixed effect	*b*	*SE*	*t*/*z*
General memory	Intercept	**0.55**	**0.02**	**32.54**
	Retraction	0.02	0.01	1.60
	Reminder	− 0.01	0.01	− 0.52
	Reading proficiency	**0.05**	**0.01**	**3.61**
	Verbal WM	0.00	0.01	0.14
	Nonverbal WM	− 0.00	0.01	− 0.36
	Retraction * Reading proficiency	0.00	0.01	0.16
	Reminder * Reading proficiency	− 0.00	0.02	− 0.13
	Retraction * Verbal WM	0.01	0.01	0.70
	Reminder * Verbal WM	0.02	0.02	1.26
	Retraction * Nonverbal WM	− 0.01	0.01	− 0.67
	Reminder * Nonverbal WM	0.01	0.01	0.36
Critical cause	Intercept	**0.72**	**0.31**	**2.33**
	Retraction	**0.82**	**0.2**	**4.01**
	Reminder	− **0.56**	**0.23**	− **2.44**
	Reading proficiency	**0.46**	**0.14**	**3.33**
	Verbal WM	− 0.18	0.12	− 1.45
	Nonverbal WM	0.05	0.12	0.43
	Retraction * Reading proficiency	0.25	0.24	1.05
	Reminder * Reading proficiency	0.26	0.27	0.95
	Retraction * Verbal WM	0.05	0.21	0.22
	Reminder * Verbal WM	− 0.29	0.25	− 1.18
	Retraction * Nonverbal WM	0.19	0.22	0.88
	Reminder * Nonverbal WM	− 0.16	0.24	− 0.66
Alternative cause	Intercept	**1.09**	**0.23**	**4.81**
	Reminder	− **0.70**	**0.29**	− **2.40**
	Reading proficiency	**0.58**	**0.17**	**3.38**
	Verbal WM	0.13	0.15	0.88
	Nonverbal WM	− 0.12	0.14	− 0.86
	Reminder * Reading proficiency	− 0.41	0.29	− 1.42
	Reminder * Verbal WM	− 0.08	0.24	− 0.34
	Reminder * Nonverbal WM	− 0.13	0.24	− 0.56
Retraction	Intercept	− 0.34	0.24	− 1.44
	Reminder	− **1.02**	**0.22**	− **4.64**
	Reading proficiency	**0.69**	**0.19**	**3.71**
	Verbal WM	− 0.02	0.17	− 0.11
	Nonverbal WM	− 0.06	0.16	− 0.36
	Reminder * Reading proficiency	− 0.12	0.27	− 0.44
	Reminder * Verbal WM	− 0.20	0.24	− 0.81
	Reminder * Nonverbal WM	− 0.25	0.24	− 1.07
Inference	Intercept	**0.58**	**0.03**	**17.52**
	Retraction	**0.18**	**0.04**	**4.12**
	Reminder	**0.06**	**0.03**	**2.08**
	Reading proficiency	− **0.04**	**0.01**	− **3.00**
	Verbal WM	− 0.01	0.01	− 0.52
	Nonverbal WM	0.00	0.01	0.29
	Retraction * Reading proficiency	**0.06**	**0.02**	**3.12**
	Reminder * Reading proficiency	0.04	0.02	1.48
	Retraction * Verbal WM	0.02	0.02	0.93
	Reminder * Verbal WM	0.03	0.02	1.17
	Retraction * Nonverbal WM	− 0.01	0.02	− 0.76
	Reminder * Nonverbal WM	− 0.04	0.02	− 1.93

Significant effects are bolded. Because questionnaire data was collected for all 88 participants prior to exclusions, additional models using this larger participant sample with no individual difference predictors were tested. These analyses revealed an identical pattern of significance for the retraction and reminder effects to the main analyses reported

General memory scores showed no significant effects of retraction or reminder (|*t*|s < 1.60), indicating equivalent memory for general details across the three conditions. Recall of the original critical cause showed a significant retraction effect (*z* = 4.01), reflecting higher recall of the original critical information in the NR compared to the average of the RNR and RER conditions. This effect was qualified by a significant reminder effect (*z* = − 2.44), such that recall of the critical cause was higher in the RER than in the RNR condition. Recall of the alternative cause and of the retraction itself, which were restricted to the retraction conditions, also showed significant reminder effects (|*z*|s > 2.40), with better recall in the RER than the RNR condition. Inference scores showed significant retraction and reminder effects (|*t*|s > 2.08). Inference scores were lower in the retraction conditions compared to the NR condition, with the greatest reduction observed in the RER condition.

**Fig. 1:**
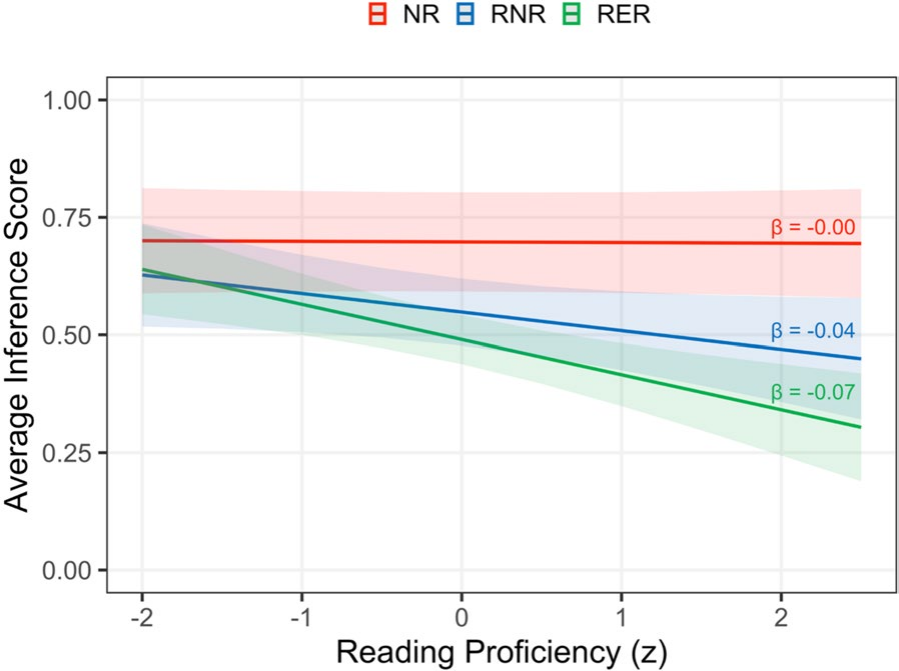
Average inference score across different levels of reading proficiency by condition. *NR* = no-retraction, *RNR* = retraction-with-no-reminder, *RER* = retraction-with-explicit-reminder

Higher reading proficiency was associated with better general memory, higher recall of the critical cause, alternative cause, and retraction, and lower inference scores (|*t*/*z*|s > 3.00). Reading proficiency also moderated the retraction effect on inference scores (*t* = 3.12): As shown in Fig. 1, individuals with higher reading proficiency showed a greater reduction in inference scores in the retraction conditions relative to the NR condition, compared to individuals with lower reading proficiency. Verbal and nonverbal WM capacity did not significantly predict any questionnaire measure (|*t*/*z*|s < 1.45) and did not interact with the retraction or reminder effects (|*t*/*z*|s < 1.93).

In summary, the questionnaire results revealed no difference in memory for general details across the three conditions, but recall of the original critical information was higher for the control NR condition than for the two retraction conditions. Explicit reminders of the original misinformation enhanced recall of the critical cause, alternative cause, and retraction. Inference scores reflecting misinformation susceptibility were also reduced when the retraction of the original misinformation included an explicit reference back to it. Finally, higher reading proficiency was associated with better overall text comprehension and reduced susceptibility to retracted information, but there were no reliable effects of WM capacity.

### Eye-movement analyses

Eye-movement data were preprocessed following standard procedures (Eskenazi, 2023; Schotter & Dillon, 2025). Adjacent fixations shorter than 60 ms that were within one-letter space were merged (0.41% of total fixations), and remaining fixations shorter than 60 ms or longer than 1,200 ms were removed (1.38% of the data). Two sets of eye-movement measures were computed: *global* reading measures for each passage of each scenario, and *local* reading measures for predefined regions of interest in the second passage, described below. As for the questionnaire analyses, both global and local measures were analyzed using GLMM/LMMs testing fixed effects of condition, individual-difference predictors, and their interactions, unless otherwise specified. Criteria for the random-effects structures and significance thresholds were identical to those used in the questionnaire analyses.

#### Global reading measures

Three global reading measures were computed for each passage: (i) *total fixation count*; (ii) *total passage duration*; and (iii) *average reading speed*, as indexed by *words per minute* (*WPM*). Total passage duration and WPM showed ex-Gaussian distributions and were log-transformed prior to analysis (Baayen, 2008). For analyses of the second passage, which differed across conditions, models controlled for passage length. Summary data are reported in Table 3, and full model outputs are provided in Table 4.

**Table 3:** Mean (and standard deviation) global reading measures for both passages by condition

		NR	RNR	RER
First passage	Total fixation count	122.72 (6.05)	120.79 (10.49)	121.30 (10.51)
	Total passage duration (ms)	32,016.04 (1408.26)	31,573.02 (2546.22)	31,649.48 (2591.88)
	WPM	193.66 (11.56)	196.63 (13.2)	197.8 (15.08)
Second passage	Total fixation count	89.18 (17.29)	116.55 (12.1)	127.85 (12.86)
	Total passage duration (ms)	23,187.52 (4505.59)	30,355.41 (3108.08)	33,136.75 (3521.9)
	WPM	211.02 (16.09)	209.71 (14.6)	218.98 (16.61)

The average length of the first passage was 100.42 words (*SD* = 17.98), which was identical across the conditions. The average length of the second passage was 77.33 words (*SD* = 19.56) in the NR condition, 100.08 words (*SD* = 18.66) in the RNR condition, and 113.83 words (*SD* = 20.38) in the RER condition

**Table 4:** Results of the (G)LMMs for the global reading measures for both passages

		First passage			Second passage		
	Fixed effect	*b*	*SE*	*t*/*z*	*b*	*SE*	*t*/*z*
Total fixation count	Intercept	**4.78**	**0.04**	**106.87**	**3.78**	**0.06**	**66.60**
	Retraction	**0.01**	**0.01**	**1.98**	− 0.05	0.03	− 1.74
	Reminder	0.00	0.01	0.14	0.03	0.02	1.72
	Reading proficiency	− 0.00	0.02	− 0.18	− 0.02	0.03	− 0.96
	Verbal WM	− 0.02	0.02	− 0.94	− 0.03	0.02	− 1.28
	Nonverbal WM	0.01	0.02	0.34	− 0.00	0.02	− 0.05
	Length	–	–	–	**0.01**	**0.00**	**17.28**
	Retraction * Reading proficiency	− 0.01	0.01	− 1.24	0.01	0.01	1.23
	Reminder * Reading proficiency	− 0.00	0.01	− 0.04	0.01	0.01	1.11
	Retraction * Verbal WM	0.01	0.01	0.92	− 0.01	0.01	− 1.04
	Reminder * Verbal WM	0.00	0.01	0.37	0.01	0.01	1.07
	Retraction * Nonverbal WM	− 0.01	0.01	− 0.80	0.00	0.01	0.51
	Reminder * Nonverbal WM	0.01	0.01	0.63	− 0.01	0.01	− 0.70
Total passage duration	Intercept	**10.34**	**0.04**	**237.4**	**9.40**	**0.07**	**136.91**
	Retraction	0.01	0.01	1.84	− **0.07**	**0.03**	− **2.46**
	Reminder	0.00	0.01	0.23	0.03	0.02	1.73
	Reading proficiency	− **0.05**	**0.02**	− **2.16**	− **0.07**	**0.03**	− **2.39**
	Verbal WM	− 0.01	0.02	− 0.72	− 0.03	0.03	− 1.09
	Nonverbal WM	0.00	0.02	0.05	− 0.00	0.02	− 0.19
	Length	–	–	–	**0.01**	**0.00**	**12.78**
	Retraction * Reading proficiency	− 0.00	0.01	− 0.46	0.01	0.01	0.95
	Reminder * Reading proficiency	0.01	0.01	0.45	0.00	0.02	0.04
	Retraction * Verbal WM	0.00	0.01	0.36	− 0.00	0.01	− 0.32
	Reminder * Verbal WM	0.00	0.01	0.00	0.00	0.01	0.34
	Retraction * Nonverbal WM	− 0.01	0.01	− 0.85	− 0.00	0.01	− 0.28
	Reminder * Nonverbal WM	0.00	0.01	0.09	0.00	0.01	0.14
WPM	Intercept	**5.26**	**0.02**	**218.93**	**5.28**	**0.06**	**87.26**
	Retraction	− 0.01	0.01	− 1.39	0.00	0.02	0.09
	Reminder	− 0.00	0.01	− 0.20	− **0.03**	**0.01**	− **2.26**
	Reading proficiency	**0.05**	**0.02**	**2.14**	**0.07**	**0.03**	**2.35**
	Verbal WM	0.01	0.02	0.70	0.03	0.03	1.07
	Nonverbal WM	− 0.00	0.02	− 0.07	0.00	0.02	0.15
	Length	–	–	–	0.00	0.00	1.02
	Retraction * Reading proficiency	0.00	0.01	0.48	− 0.01	0.01	− 0.94
	Reminder * Reading proficiency	− 0.00	0.01	− 0.40	0.00	0.02	0.01
	Retraction * Verbal WM	− 0.00	0.01	− 0.36	0.00	0.01	0.27
	Reminder * Verbal WM	0.00	0.01	0.00	− 0.00	0.01	− 0.11
	Retraction * Nonverbal WM	0.01	0.01	0.82	0.00	0.01	0.21
	Reminder * Nonverbal WM	− 0.00	0.01	− 0.12	− 0.00	0.01	− 0.07

Significant effects are bolded. Because eye-movement data was available for 85 participants prior to exclusions, additional models using this larger participant sample with no individual difference predictors were tested. These analyses revealed a pattern of significance for the retraction and reminder effects that was identical to the main analyses reported with the following exceptions: In the first passage, the retraction effect on total fixation count was marginally significant (*b* = 0.01, *SE* = 0.01, *z* = 1.94) and, in the second passage, the reminder effect on total fixation count was significant (*b* = 0.04, *SE* = 0.02, *z* = 2.16)

For the first passage, which was identical across conditions, there were no significant retraction or reminder effects on any measure (|*t*/*z*|s < 1.84), with the exception of slightly more fixations in the NR condition than the average of the two retraction conditions (*z* = 1.98). For the second passage, a significant retraction effect emerged on total passage duration (*t* = − 2.46), reflecting shorter reading times in the NR condition than in the average of the RNR and RER conditions. There was also a significant reminder effect observed on WPM (*t* = − 2.26), with faster reading speeds in the RER than in the RNR condition.

Across both passages, higher reading proficiency was associated with more efficient reading behavior, including shorter passage durations and faster WPM (|*t*|s > 2.14). Reading proficiency did not interact with either the retraction or reminder effect on any global measure (|*t*/*z*|s < 1.24). Verbal and nonverbal WM capacity were not significant predictors of any global measure (|*t*/*z*|s < 1.28) and they did not interact with the retraction or reminder effects (|*t*/*z*|s < 1.07).

In summary, there was little evidence that global reading measures differed across conditions for the first passage, as expected given that the content was identical. However, for the second passage, there was some indication of longer reading times when a retraction was present, and faster reading speeds when the retraction included an explicit reminder, even after controlling for passage length. More generally, across both passages, higher reading proficiency was associated with more efficient text processing, but there were no effects of WM capacity.

#### Local reading measures

**Fig. 2:**
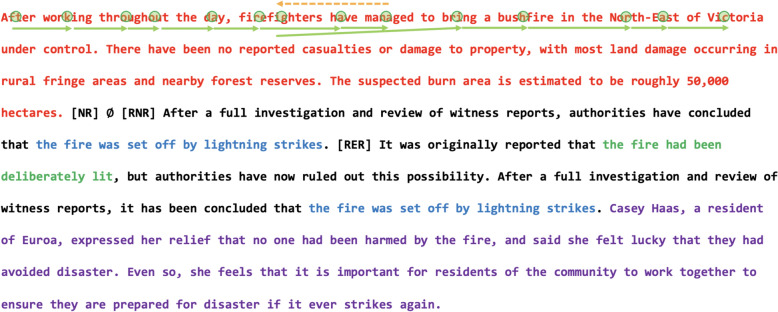
An example passage with the opening region in red, the closing region in purple, the alternative region in blue, and the critical region in green. The first line illustrates how eye movements may have progressed along the text: green circles indicate fixations, green solid lines indicate forward saccades, and orange dotted lines indicate backward saccades or regressions. Words without a fixation were skipped. *NR* = no-retraction, *RNR* = retraction-with-no-reminder, *RER* = retraction-with-explicit-reminder

Three local reading measures were computed for four predefined regions of interest in the second passage of each scenario. The *opening region* preceded any mention of the original critical or alternative causal information and contained only non-causal details about the event. The *closing region* followed any mention of causal information, and likewise also contained only non-causal details about the event. As illustrated in Fig. 2, both regions were identical across conditions. The *alternative region* contained information about the alternative cause of the event and appeared only in the RNR and RER conditions. The *critical region* contained information about the original critical cause of the event and appeared only in the RER condition.

For each word within the four regions, three local eye-movement measures were analyzed: (i) *first-pass duration* (i.e., the sum of all fixations on a word before the eyes exited the word for the first time)*,* indexing the initial stages of lexical processing; (ii) *second-pass duration* (i.e., the sum of all refixations on a word after the eyes had exited the word), indexing post-lexical processing and rereading; and (iii) *total duration* (i.e., the sum of all fixations during first- and second-pass reading). Each of these three measures were summed across words within each region to provide a measure of its processing. Because all measures showed ex-Gaussian distributions, they were log-transformed prior to analysis (Baayen, 2008). Summary data are reported in Table 5, and full model outputs are provided in Tables 6, 7.^2^

**Table 5:** Mean (and standard deviation) local reading measures on the four regions of interest in the second passage by condition

		NR	RNR	RER
Opening	First-pass duration (ms)	4562.18 (1452.55)	4852.55 (1305.11)	4633.09 (1332.34)
	Second-pass duration (ms)	2258.93 (784.55)	2177.07 (778.98)	2042.13 (711.42)
	Total duration (ms)	10,072.73 (2725.3)	10,068.77 (2233.26)	9494.64 (1829.8)
Closing	First-pass duration (ms)	4645.37 (763.62)	4699.77 (1169.44)	4619.18 (1187.70)
	Second-pass duration (ms)	2009.00 (418.03)	1984.09 (792.64)	1742.85 (776.58)
	Total duration (ms)	9166.10 (1042.22)	8978.41 (2223.26)	8466.50 (2378.04)
Alternative	First-pass duration (ms)	–	1421.07 (688.08)	1400.93 (657.23)
	Second-pass duration (ms)	–	684.17 (522.79)	591.20 (467.12)
	Total duration (ms)	–	2911.74 (1365.56)	2823.85 (1303.51)
Critical	First-pass duration (ms)	–	–	1087.97 (749.54)
	Second-pass duration (ms)	–	–	460.82 (441.97)
	Total duration (ms)	–	–	2136.79 (1181.77)

**Table 6:** Results of the (G)LMMs for the local reading measures on the opening and closing regions in the second passage

		Opening region			Closing region		
	Fixed effect	*b*	*SE*	*t*	*b*	*SE*	*t*
First-pass duration	Intercept	**8.28**	**0.11**	**75.84**	**8.30**	**0.11**	**76.18**
	Retraction	− 0.01	0.05	− 0.16	− 0.04	0.03	− 1.26
	Reminder	0.06	0.06	0.93	− 0.02	0.04	− 0.52
	Reading proficiency	− **0.15**	**0.05**	− **3.01**	− **0.13**	**0.03**	− **4.03**
	Verbal WM	0.05	0.04	1.07	0.04	0.03	1.44
	Nonverbal WM	0.01	0.04	0.16	0.04	0.03	1.20
	Retraction * Reading proficiency	0.02	0.05	0.44	0.00	0.03	0.08
	Reminder * Reading proficiency	− 0.09	0.08	− 1.13	− 0.05	0.05	− 1.03
	Retraction * Verbal WM	− 0.06	0.05	− 1.22	0.00	0.03	0.06
	Reminder * Verbal WM	− 0.02	0.07	− 0.33	0.03	0.04	0.60
	Retraction * Nonverbal WM	0.05	0.05	0.93	− 0.06	0.03	− 1.85
	Reminder * Nonverbal WM	0.04	0.07	0.57	0.04	0.04	0.95
Second-pass duration	Intercept	**7.38**	**0.12**	**60.23**	**7.27**	**0.12**	**58.61**
	Retraction	0.08	0.04	1.88	**0.14**	**0.04**	**3.18**
	Reminder	0.03	0.10	0.28	0.08	0.09	0.94
	Reading proficiency	− 0.05	0.07	− 0.73	− 0.01	0.07	− 0.20
	Verbal WM	− 0.11	0.07	− 1.64	− 0.06	0.06	− 0.94
	Nonverbal WM	− 0.03	0.06	− 0.41	− 0.05	0.06	− 0.91
	Retraction * Reading proficiency	− 0.01	0.05	− 0.11	0.02	0.05	0.32
	Reminder * Reading proficiency	0.10	0.08	1.28	0.06	0.09	0.66
	Retraction * Verbal WM	0.01	0.05	0.22	− 0.01	0.05	− 0.20
	Reminder * Verbal WM	0.00	0.07	0.01	− 0.02	0.08	− 0.27
	Retraction * Nonverbal WM	− **0.10**	**0.05**	− **2.12**	**0.09**	**0.05**	**2.05**
	Reminder * Nonverbal WM	− 0.04	0.07	− 0.55	0.08	0.08	0.99
Total duration	Intercept	**9.09**	**0.11**	**85.06**	**8.97**	**0.11**	**80.11**
	Retraction	**0.04**	**0.02**	**2.49**	**0.07**	**0.02**	**3.61**
	Reminder	0.03	0.02	1.53	0.02	0.04	0.52
	Reading proficiency	− **0.07**	**0.03**	− **2.37**	− **0.07**	**0.03**	− **2.16**
	Verbal WM	− 0.04	0.03	− 1.62	− 0.01	0.03	− 0.20
	Nonverbal WM	− 0.01	0.02	− 0.31	− 0.01	0.03	− 0.18
	Retraction * Reading proficiency	0.01	0.02	0.30	0.00	0.02	0.03
	Reminder * Reading proficiency	0.03	0.03	1.11	0.02	0.03	0.65
	Retraction * Verbal WM	− 0.01	0.02	− 0.59	− 0.02	0.02	− 1.02
	Reminder * Verbal WM	− 0.02	0.02	− 0.73	0.04	0.03	1.29
	Retraction * Nonverbal WM	0.02	0.02	1.05	0.02	0.02	0.76
	Reminder * Nonverbal WM	− 0.00	0.02	− 0.11	0.04	0.03	1.23

Significant effects are bolded. Because eye-movement data was available for 85 participants prior to exclusions, additional models using this larger participant sample with no individual difference predictors were tested. These analyses revealed a pattern of significance for the retraction and reminder effects that was identical to the main analyses reported with the exception that the retraction effect was significant on second-pass duration in the opening region (*b* = 0.09, *SE* = 0.04, *t* = 2.21)

**Table 7:** Results of the (G)LMMs for the local reading measures on the alternative and critical regions in the second passage

		Alternative region			Critical region		
	Fixed effect	*b*	*SE*	*t*	*b*	*SE*	*t*
First-pass duration	Intercept	**7.11**	**0.14**	**50.14**	**6.88**	**0.12**	**57.62**
	Reminder	0.03	0.06	0.53	–	–	–
	Reading proficiency	− **0.14**	**0.03**	− **4.28**	− **0.14**	**0.04**	− **3.23**
	Verbal WM	0.02	0.03	0.75	− 0.05	0.04	− 1.21
	Nonverbal WM	**0.06**	**0.03**	**2.05**	0.07	0.04	1.70
	Reminder * Reading proficiency	0.07	0.04	1.49	–	–	–
	Reminder * Verbal WM	− 0.05	0.04	− 1.14	–	–	–
	Reminder * Nonverbal WM	0.00	0.04	0.13	–	–	–
Second-pass duration	Intercept	**6.41**	**0.11**	**57.88**	**6.19**	**0.06**	**105.23**
	Reminder	0.10	0.08	1.28	–	–	–
	Reading proficiency	− 0.06	0.05	− 1.09	0.01	0.07	0.20
	Verbal WM	− 0.04	0.05	− 0.84	− **0.13**	**0.06**	− **2.03**
	Nonverbal WM	0.01	0.05	0.11	0.08	0.06	1.41
	Reminder * Reading proficiency	0.07	0.09	0.78	–	–	–
	Reminder * Verbal WM	− 0.02	0.09	− 0.18	–	–	–
	Reminder * Nonverbal WM	0.03	0.08	0.41	–	–	–
Total duration	Intercept	**7.73**	**0.17**	**44.42**	**7.52**	**0.09**	**80.40**
	Reminder	0.05	0.04	1.21	–	–	–
	Reading proficiency	− 0.04	0.04	− 1.14	− 0.06	0.05	− 1.23
	Verbal WM	− **0.07**	**0.03**	− **2.12**	− **0.09**	**0.04**	− **2.08**
	Nonverbal WM	0.03	0.03	1.03	**0.10**	**0.04**	**2.42**
	Reminder * Reading proficiency	− 0.03	0.05	− 0.63	–	–	–
	Reminder * Verbal WM	0.00	0.04	0.08	–	–	–
	Reminder * Nonverbal WM	− 0.01	0.04	− 0.13	–	–	–

Significant effects are bolded. For the alternative region, because eye-movement data was available for 85 participants prior to exclusions, additional models using this larger participant sample with no individual difference predictors were tested. These analyses revealed an identical pattern of significance for the retraction and reminder effects to the main analyses reported

In the opening region, there was a significant retraction effect on total duration (*t* = 2.49), with shorter reading times in the average of the two retraction conditions than in the NR condition. No reminder effects were observed on any measure (|*t*|s < 1.53). In the closing region, significant retraction effects were observed on second-pass and total duration measures (|*t*|s > 3.18), with shorter reading times in the average of the two retraction conditions than in the NR condition. The reminder effect was not significant on any measure (|*t*|s < 1). In the alternative region, which appeared only in the RNR and RER conditions, no reminder effects were observed on any measure (|*t*|s < 1.28). Because the critical region appeared only in the RER condition, retraction and reminder effects were not tested for this region.

**Fig. 3:**
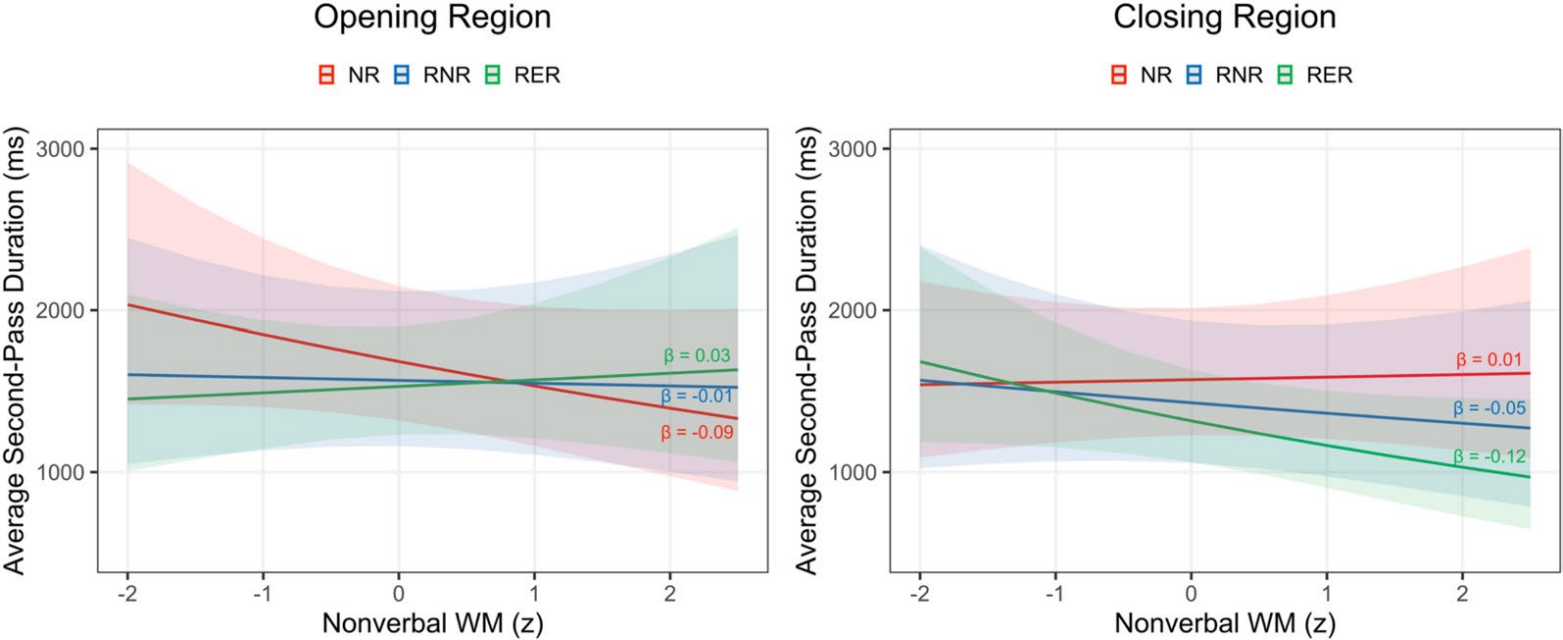
Average second-pass duration on the opening region (left panel) and closing region (right panel) of the second passage across different levels of nonverbal WM capacity by condition. *NR* = no-retraction, *RNR* = retraction-with-no-reminder, *RER* = retraction-with-explicit-reminder

Higher reading proficiency was associated with shorter first-pass durations across all regions (|*t*|s > 3.01) and with shorter total durations in the opening and closing regions (|*t*|s > 2.16). Reading proficiency did not interact with either the retraction or reminder effect on any measure (|*t*|s < 1.49). Higher verbal WM capacity was associated with shorter second-pass durations in the critical region and shorter total durations in the alternative and critical regions (|*t*|s > 2.03). Verbal WM capacity did not interact with either the retraction or reminder effect on any measure (|*t*|s < 1.29). In contrast, higher nonverbal WM capacity was associated with longer first-pass durations in the alternative region and longer total durations in the critical region (|*t*|s > 2.05). In the opening and closing regions, nonverbal WM capacity moderated the retraction effect on second-pass duration (|*t*|s > 2.05). As shown in the left panel of Fig. 3, as nonverbal WM capacity increased, second-pass reading times in the opening region decreased in the NR condition but remained stable in the two retraction conditions. As shown in the right panel of Fig. 3, as nonverbal WM capacity increased, second-pass reading times in the closing region decreased in the two retraction conditions but remained stable in the NR condition.

In summary, both the opening and closing regions of the second passage yielded shorter reading times in the two retraction conditions compared to the control NR condition. There was no evidence that local reading times differed as a function of whether there was a reminder of the original misinformation. Across regions, higher reading proficiency was associated with shorter reading times. Within regions containing causal information, higher verbal WM capacity was associated with shorter reading times, while higher nonverbal WM capacity was associated with longer reading times. Higher nonverbal WM capacity was linked to less re-reading in the opening region of the NR condition and in the closing region of the retraction conditions.

#### Predicting the CIE from eye movements

Two supplementary analyses were conducted to examine whether individuals’ eye movement behavior during the processing of misinformation predicted their subsequent susceptibility to the continued influence of retracted information. These analyses tested whether the way participants encode information translates into the persistence of false or inaccurate beliefs, a relationship that has not yet been systematically investigated in previous research. LMMs were conducted to predict offline inference scores, which most directly capture the CIE, from online reading measures that were shown to differ across conditions in the primary analyses. Models 1 and 2 included the global reading measures of total passage duration and WPM for the second passage of each scenario, controlling for passage length. Model 3 included local reading measures of total duration in the opening and closing regions of the second passage. The effect of condition on these predictive relationships was also tested. Full model outputs are provided in Table 8.^3^

**Table 8:** Results of the LMMs using eye-movement measures to predict the CIE

Model	Fixed effect	*b*	*SE*	*t*
1	Intercept	**0.47**	**0.13**	**3.55**
	Retraction	**0.19**	**0.06**	**3.51**
	Reminder	**0.09**	**0.03**	**3.29**
	Total passage duration	− 0.01	0.01	− 0.79
	Length	0.00	0.00	0.77
	Retraction * Total passage duration	− 0.03	0.02	− 1.44
	Reminder * Total passage duration	− 0.04	0.02	− 1.95
2	Intercept	**0.41**	**0.11**	**3.60**
	Retraction	**0.22**	**0.05**	**4.13**
	Reminder	**0.08**	**0.03**	**2.53**
	WPM	0.01	0.01	0.83
	Length	0.00	0.00	1.53
	Retraction * WPM	0.01	0.01	0.82
	Reminder * WPM	**0.04**	**0.02**	**2.27**
3	Intercept	**0.58**	**0.03**	**18.21**
	Retraction	**0.17**	**0.04**	**4.42**
	Reminder	**0.05**	**0.02**	**3.17**
	Total duration opening region	0.00	0.01	0.23
	Total duration closing region	− 0.01	0.01	− 0.71
	Retraction * Total duration opening region	− 0.02	0.02	− 1.46
	Reminder * Total duration opening region	− 0.03	0.02	− 1.67
	Retraction * Total duration closing region	0.00	0.02	0.28
	Reminder * Total duration closing region	− **0.04**	**0.02**	− **2.62**

Significant effects are bolded. The eye-movement measures used as predictors were total passage duration (Model 1), WPM (Model 2) and total duration in the opening and closing regions (Model 3)

**Fig. 4:**
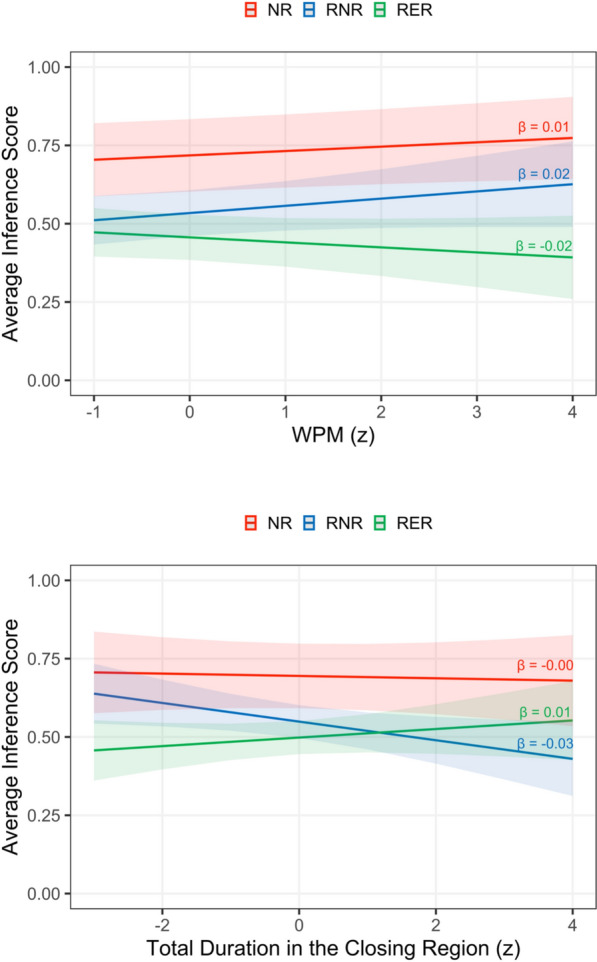
Average inference score predicted by WPM (upper panel), and total duration in the closing region (lower panel) by condition. *NR* = no-retraction, *RNR* = retraction-with-no-reminder, *RER* = retraction-with-explicit-reminder

Across all models, the retraction and reminder effects on inference scores remained significant (|*t*|s > 2.53), consistent with the primary analyses. None of the global (total passage duration and WPM) or local (total duration in the opening and closing regions) reading measures significantly predicted inference scores (|*t*|s < 1). However, there were significant interactions between the reminder effect and both WPM and total duration in the closing region (|*t*|s > 2.27). As shown in the upper and lower panels of Fig. 4, slower overall reading speeds and longer reading times in the closing region were associated with reduced inference scores in the RNR condition but increased inference scores in the RER condition. Saying this another way, longer processing times were linked to a smaller benefit from the explicit reminder compared to shorter processing times.

These supplementary analyses provide tentative evidence of a link between online reading behavior and subsequent misinformation susceptibility because inference scores in the retraction conditions were predicted by different reading patterns depending on whether there was a reminder of the original misinformation. Longer reading times in the closing region of the second passage, as well as slower reading speeds across the entire passage, were associated with reduced susceptibility to misinformation when no explicit reminder was provided, but with increased susceptibility to misinformation when the retraction included a reminder.

## Discussion

The *continued-influenced effect* (*CIE*) of misinformation is a robust phenomenon observed across a number of studies (see Lewandowsky et al., 2012; Walter & Tukachinsky, 2020 for reviews). Using novel scenarios about fictional news events, the present study replicated this effect by showing that information initially presented as true continued to influence individuals’ inferential judgments even after it was retracted. However, consistent with studies that have focused on strategies to reduce misinformation susceptibility (Ecker et al., 2017; Xu et al., 2020), this influence was significantly reduced when there was an explicit reminder of the original critical information. The ability to explicitly recall the critical cause, alternative cause, and retraction was also better when a reminder was present. These findings provide further evidence against the ‘familiarity backfire effect’ which posits that repeating misinformation enhances its familiarity and therefore believability (Lewandowsky et al., 2012; Pluviano et al., 2017; Swire et al., 2017). Instead, they support the view that reminders highlight the discrepancy between the causal explanations provided, allowing individuals to update their understanding of the event by replacing old incorrect information with new correct information (Ecker et al., 2017; Kendeou et al., 2014; Putnam et al., 2014).

The pattern of recalling the critical and alternative causes observed in the current study, however, was subtly different from that of previous studies (Ecker et al., 2017; Xu et al., 2020). Ecker et al. (2017) originally reported no difference in recall of the critical cause between retraction and no retraction passages. Xu et al. (2020) subsequently found a difference, such that recall of the critical cause was better in passages that contained a retraction than in those that did not, which they attributed to time constraints encouraging participants to focus on those details in the retraction conditions. The present results, however, indicated that the critical cause was recalled better in passages containing no retraction compared to passages containing a retraction. A similar discrepancy was observed for recall of the alternative cause between the two retraction conditions: Whereas both Ecker et al. and Xu et al. reported no difference between the conditions, the present results indicated that recall of the alternative cause was better when retraction of the original critical information was accompanied by an explicit reminder.

These differences in recall of the critical and alternative causes likely reflect the consequences of a key methodological aspect of the current research. Whereas Ecker et al. and Xu et al. presented six and four scenarios, respectively, followed by a distractor task and then questionnaires, the present experiment presented three blocks of four scenarios, with each block followed immediately by a set of questionnaires. Thus, the current design not only provided greater statistical power due to the increased number of scenarios, but may have also alerted participants to the comprehension requirements after the first block, thereby encouraging greater attention to information about the causes of events in subsequent blocks. Consistent with this possibility, average recall of the critical and alternative causes in the present experiment was 64% and 71%, respectively, compared to the lower scores reported by both Ecker et al. (52%, 41%) and Xu et al. (55%, 55%).^4^ The time constraints imposed on each passage may have further encouraged participants to focus on the critical and alternative causal information at the expense of non-causal details, which is consistent with the finding that memory for general details was equivalent across conditions.

The eye-movement data collected while individuals read these texts provide complementary insight into the real-time processing of misinformation. Both global measures of the entire second passage and local measures of specific regions of the second passage indicated that individuals were sensitive to changes in causal information. At the global level, passages containing a retraction of the original critical information yielded longer overall reading times than passages containing no retraction. With passage length controlled for, this finding likely reflects the increased processing effort associated with encoding corrective information. The local eye-movement data provide further support for this interpretation, as the opening and closing regions of the retraction passages received shorter total reading times than those of the no-retraction passages, with a similar reduction also observed on second-pass reading times in the closing region. Because these opening and closing regions contained non-causal details, reduced engagement with these regions in the retraction conditions suggests that participants may have strategically allocated more time to processing the transition text, which contained the updated causal information. Given that there was no transition text in the NR condition, and that the transition text was longer in the RER than RNR condition due to the inclusion of a reminder, this strategic allocation of attention is especially plausible in light of the time constraints imposed on the reading of each passage. Importantly, the reduced reading times in the opening and closing regions of the retraction passages did not lead to poorer memory for non-causal details about the event, as indicated by the offline questionnaire data. Together, these findings from both global- and local-level processing of the second passage are consistent with previous eye-movement studies (e.g., Kim et al., 2021; Lee et al., 2024; Wellons & Wahlheim, 2025) in showing that individuals are sensitive to the presence of misinformation. Specifically, misinformation increases processing effort due to the encoding of corrective information while simultaneously reducing reading of non-causal details.

There was also some indication from the eye-tracking data that individuals were sensitive to reminder-based correction strategies, consistent with the improved recall and inferential judgements observed in the questionnaire data. The clearest evidence came from global-level processing of the second passage, which showed faster overall reading speeds when retractions included a reminder of the original misinformation compared to when they did not. This likely reflects that these retraction passages were easier to understand when the alternative cause was presented alongside an explicit reminder of the original cause. Supplementary analyses further indicated that faster reading speeds in the retraction passages with a reminder were associated with lower inference scores, reflecting reduced misinformation susceptibility. Notably, however, there was no evidence from local-level processing to indicate sensitivity to reminder-based corrections of misinformation. Both early and late eye-movement measures were equivalent in regions containing information about the alternative cause of the event, irrespective of whether there was also an explicit reminder of the original cause. These comparable effects also extended to the opening and closing regions of the retraction passages. Together, these findings suggest that reminder-based corrections may facilitate reading by easing memory encoding across the entire passage rather than by affecting processing of specific regions. More generally, they are compatible with the view that reminders are beneficial for correcting misinformation because they facilitate integration of updated information (Ecker et al., 2017; Kendeou et al., 2014; Putnam et al., 2014).

Individual differences in cognitive abilities further moderated the effects of misinformation in both offline and online measures. Reading proficiency was linked to better overall text comprehension and reduced susceptibility to misinformation in the questionnaire data. Reading proficiency also modulated the retraction effect on inference scores because skilled readers were less susceptible to retracted misinformation. Because reading proficiency did not interact with the reminder effect, this resistance appeared to occur even when there was no explicit reference to the original critical information. Skilled readers therefore appear better able to reconcile conflicting information and construct a veridical and coherent mental representation of the situation with minimal repetition, even when rereading earlier information is not possible.

Eye-movement data further revealed that reading proficiency was associated with more efficient global reading patterns, consistent with prior research using naturalistic texts (e.g., Andrews et al., 2022). This facilitation extended to local processing of the second passage, with skilled readers showing faster first-pass reading across all regions, including those containing information related to the original and updated cause of the event. Notably, reading proficiency did not further modulate retraction or reminder effects on eye-movement measures in the second passage. Taken together, these findings extend previous research (De Keersmaecker & Roets, 2017; Xu et al., 2020) by demonstrating a link between reading proficiency, indexed by both vocabulary and comprehension, and misinformation susceptibility.

In contrast, although WM capacity predicted online eye-movement measures, it did not predict offline text comprehension or susceptibility to misinformation. This finding is somewhat surprising given that the capacity to actively maintain and manipulate task-relevant information has been linked to comprehension (Daneman & Carpenter, 1980; Daneman & Merikle, 1996) and the ability to generate inferences from written text (Linderholm, 2002; Singer et al., 1992). However, our data is consistent with evidence that verbal WM capacity does not predict reading comprehension when reading-related skills are controlled (Van Dyke et al., 2014), and with mixed findings in the misinformation literature (e.g., Brydges et al., 2018; Sanderson et al., 2021). These results provide further evidence against integration-based accounts of the CIE, which attribute misinformation susceptibility to failures in encoding and integrating a correction with the original information in memory (Johnson & Seifert, 1994; Wilkes & Leatherbarrow, 1988). It should be noted, however, that the results do not necessarily support retrieval-based accounts of the CIE, which posit that misinformation persists due to automatic familiarity processes (Ecker et al., 2010b; Lewandowsky et al., 2012; Swire et al., 2017), as there was no evidence that the repetition of misinformation increased the CIE.

Despite the absence of offline effects, WM capacity influenced local eye-movement behavior in specific regions of the second passage. Higher verbal WM was associated with shorter reading times on late measures in regions containing causal information. Processing of this information therefore appeared to be more efficient for individuals with larger verbal WM capacity, consistent with the view that they are more skilled at the simultaneous maintenance and manipulation of verbal information, even though this did not appear to lead to better subsequent recall of this information or reduced misinformation susceptibility, as measured by the offline questionnaire data.

Interestingly, nonverbal WM capacity was also a significant predictor of reading patterns in both the critical and alternative regions, but in the opposite direction to what would be expected: Higher nonverbal WM capacity was associated with longer reading of information related to the original and updated cause of the event. This finding suggests that a larger nonverbal WM span does not facilitate the processing of causal information that is verbal in nature. Furthermore, in the opening and closing regions, nonverbal WM capacity modulated the processing of retracted information such that higher nonverbal WM capacity was associated with shorter rereading times in the opening region of the passages without a retraction and in the closing region of the passages with a retraction. Although a straightforward interpretation of these patterns is difficult, it does appear that the ability to store and manipulate nonverbal information predicts processing of non-causal details about an event. Taken together, these findings suggest that individuals’ ability to hold verbal information in WM facilitates real-time processing of causal information, whereas their ability to hold nonverbal information predicts real-time processing of more peripheral information. These findings likely reflect the fact that the reading task is, by definition, verbal in nature, and that verbal and nonverbal memory processes are likely to be independent (Friedman & Miyake, 2000; Shah & Miyake, 1996).

The final contribution of the present research was to evaluate whether misinformation susceptibility could be directly predicted from online eye-movement behavior. The findings revealed that both global- and local-level processing of the second passage predicted subsequent inference scores in the retraction passages, depending on whether a reminder of the original misinformation was present. Specifically, when individuals showed slower reading speeds across the entire passage, they subsequently showed lower inference scores, reflecting a weaker CIE, in the RNR condition, but higher inferences, reflecting a stronger CIE, in the RER condition. A similar pattern emerged for processing times in the closing region of the second passage: As reading times increased, inference scores decreased in the RNR condition but increased in the RER condition.

Assuming that slower reading speeds and longer reading times reflect more effortful processing, the patterns associated with lower inference scores in the RNR condition may reflect individuals updating their overall understanding of the event with the new corrected information. Somewhat surprisingly though, these patterns did not appear to produce the same updating processes in the RER condition, which was associated with higher inference scores despite the availability of an explicit reminder of the original misinformation. In this case, slower reading speeds and/or longer reading times in the RER condition may reflect more effortful processing arising from a failure to integrate the two causal explanations of the event, resulting in a stronger CIE. These findings therefore suggest a tentative link between individuals’ eye-movement behavior during misinformation processing and the persistence of false or inaccurate beliefs. Future research could provide additional insight by employing more tightly controlled designs to directly examine the relationship between misinformation susceptibility and eye-movement behavior in regions containing original and/or new causal information.

In summary, the offline questionnaire replicated prior findings by showing that the CIE was reduced when the original critical information was explicitly repeated at the time of retraction. Eye-movement data further revealed that retractions of causal information were associated with increased overall processing effort due to the encoding of corrective information, and reduced re-reading of non-causal details. However, reminder-based strategies for correcting misinformation were linked to faster overall reading speeds, suggesting that explicit references to the original misinformation facilitated integration of the updated information. Supplementary analyses revealed a tentative link between these offline and online measures, such that longer reading times and slower reading speeds predicted reduced misinformation susceptibility but only when no explicit reminder accompanied the retraction. However, further research is necessary to clarify this predictive relationship.

Finally, individual differences in cognitive abilities played a role in individuals’ sensitivity and susceptibility to misinformation, although the generalizability of these effects should be interpreted with caution given the relatively homogenous sample of young, predominantly female university students. Individuals with higher reading proficiency showed greater reductions in the CIE, although they derived minimal additional benefit from the reminder-based corrections. Higher reading proficiency and higher verbal WM capacity also facilitated real-time processing of regions containing causal information. Future research should replicate these findings in more diverse samples varying in age, gender, and educational background to assess their generalizability.

Taken together, the present findings attest to the pervasiveness of misinformation during reading, even in the context of neutral newspaper-style articles that should not bias interpretation. Notably, resistance to correction does not appear to reflect inherent misunderstanding of presented facts because individual differences in cognitive abilities, particularly reading proficiency, affected misinformation susceptibility. Nevertheless, reminder-based correction strategies appear effective for minimizing misinformation reliance in the real world by explicitly highlighting the conflict between the causal explanations provided for an event. Notably, this benefit may be attenuated for highly proficient readers.

The present research therefore extends previous investigations of the CIE by using eye-tracking to provide real-time insight into misinformation processing, the effectiveness of correction strategies, and the role of individual differences. Unlike prior studies, these insights emerged from a broader set of eye-movement measures capturing both global and local processing. Future research should continue to use eye tracking to investigate the cognitive mechanisms underlying misinformation effects.

## Data Availability

The experiment materials and de-identified data analyzed for the present research are publicly available at the Open Science Framework website: https://osf.io/2qxbw/. This research was not pre-registered.

## Footnotes

1 Prior to exclusions, each counterbalanced list was completed by 21–23 participants. After excluding 14 participants for the reasons described above, each list was completed by 17–20 participants.

2 This analytic approach was adopted instead of extracting aggregated local reading measures from each region as a single interest area for two reasons. First, defining interest areas at the word level reduces the impact of saccadic error, as inaccurate eye movements are isolated to individual words rather than affecting an entire region. Second, word-based interest areas are more precise and consistent because they are generated automatically during data preprocessing. This contrasts with region-based interest areas, which must be manually drawn, and are therefore more susceptible to human error. Additional analyses examining aggregated local reading measures extracted from region-based interest areas revealed a pattern of significance for most effects that was identical to those reported in the main text with the following exceptions. The reminder effect on first-pass duration in the alternative region (*b* = -0.20, *SE* = 0.10, *t* = -2.05) and the Reminder × Verbal WM interaction on second-pass duration in the closing region (*b* = 0.45, *SE* = 0.19, *t* = 2.41) were significant. The retraction effect on second-pass duration and the reading proficiency effect on first-pass duration were not significant in the closing region (|*t*|s < 1.06). The reading proficiency effect and the nonverbal WM capacity effect on first-pass duration were not significant in the alternative region (|*t*|s < 1). The Retraction × Nonverbal WM interaction on second-pass duration in the opening and closing regions were not significant (|*t*|s < 1).

3 Because individual differences were not tested in these models, the analyses included the data from the additional 11 participants who had been excluded from the primary analyses reported due to missing or unreliable individual differences measures. Follow-up analyses restricted to the subset of 74 participants included in the primary analyses yielded an identical pattern of significant results, except that the following effects were non-significant (|*t*|s < 1.80): the interactions between the reminder effect and WPM, and between the reminder effect and total duration on the closing region. Given the exploratory nature of these analyses, results from the larger sample of 85 participants are reported in the main text.

4 Some further evidence that comprehension demands may have changed over the course of the task comes from analyses that included trial order as a mean-centered predictor. These analyses revealed a significant effect of trial order on recall of the critical cause, which improved over the course of the task (*b* = 0.30, *SE* = 0.09, *z* = 3.43), and an increasingly efficient eye-movement pattern, e.g., fewer total fixations, faster overall speeds (|*t*/*z*|s > 3.67), and shorter durations on global and local measures (|*t*|s > 2.07). Importantly, however, when controlling for trial order, the pattern of significance across all analyses was identical to that reported in the main analyses, with two exceptions: The retraction effect on total fixation count was not significant in the first passage (*z* = 1.20) and became significant in the second passage (*b* = -0.06, *SE* = 0.03, *z* = -2.23). Thus, although the design of the current study may have partially encouraged participants to focus on certain details, thereby impacting their overall questionnaire performance, there was little evidence that these changing task demands systematically altered eye-movement behavior across conditions.
